# Implantation of Bioreactor-Conditioned Plant-Based Vascular Grafts

**DOI:** 10.3390/jfb17010043

**Published:** 2026-01-15

**Authors:** Tai Yin, Nicole Gorbenko, Christina Karras, Samantha E. Nainan, Gianna Imeidopf, Arvind Ramsamooj, Sleiman Ghorayeb, Nick Merna

**Affiliations:** 1 Feinstein Institutes for Medical Research, Northwell Health, Manhasset, NY 11030, USA; tyin@northwell.edu (T.Y.); ngorbenko1@pride.hofstra.edu (N.G.); sleiman.r.ghorayeb@hofstra.edu (S.G.); 2 School of Engineering and Applied Science, Bioengineering, Hofstra University, Hempstead, NY 11549, USA; ckarras1@pride.hofstra.edu (C.K.); samanthanainan@gmail.com (S.E.N.); gimeidopf1@pride.hofstra.edu (G.I.); aramsamooj1@pride.hofstra.edu (A.R.); 3 Hofstra-Northwell School of Medicine, Radiology and Molecular Medicine, Hempstead, NY 11549, USA

**Keywords:** small-diameter vascular graft, plant-based scaffold, decellularization, implantation, bioreactor conditioning

## Abstract

Small-diameter synthetic grafts often fail from thrombosis, intimal hyperplasia, and compliance mismatch, highlighting the need for alternatives that better support endothelialization and remodeling. Here, we evaluated multilayer plant-based vascular grafts fabricated from decellularized leatherleaf viburnum reinforced with cross-linked gelatin, seeded with vascular smooth muscle cells and endothelial cells, and conditioned in a perfusion bioreactor to mimic physiological shear stress. Pre-implant assays confirmed effective decellularization, low residual detergent, and mechanical integrity suitable for surgical handling. In a rat abdominal aorta interposition model, plant-based grafts remained patent at 1, 4, and 24 weeks and showed higher survival than silicone controls. Ultrasound imaging demonstrated flow patterns and resistance indices similar to native vessels, and plant-based grafts maintained significantly higher endothelial cell coverage than silicone controls, reaching native-like density by 24 weeks. Histology and biochemical assays showed early collagen and elastin coverage comparable to native aorta and increased collagen by 24 weeks. Scanning electron microscopy showed smooth luminal surfaces with minimal thrombus formation, contrasting with the rougher, thrombus-prone surfaces of silicone grafts. These findings indicate that plant-based grafts support endothelialization, maintain long-term patency, and undergo favorable remodeling in vivo, supporting their potential as a biomimetic alternative for small-diameter arterial repair.

## 1. Introduction

Coronary heart disease and peripheral artery disease often require arterial bypass [1,2]. Over 600,000 coronary artery bypass surgeries are conducted annually, ranking it among the most frequently performed major surgical procedures [3]. The leg’s saphenous vein is commonly used to redirect blood flow around the obstructed artery because it can be transplanted quickly in emergencies. However, 20% of patients do not have a suitable saphenous vein due to previous vein harvesting, inadequate quality, or small size [4,5].

Small-diameter (<6 mm) vascular grafts still face high failure rates due to acute thrombosis, intimal hyperplasia, and compliance mismatch [6,7]. Traditional synthetics like ePTFE, Dacron, and silicone perform well in large arteries but show poor 1-year patency (40%) in coronary-sized applications [8] due to thrombosis and intimal hyperplasia [9,10,11] despite efforts to enhance endothelialization using extracellular matrix (ECM) proteins and growth factors [12,13]. Their hydrophobic luminal surfaces inhibit endothelial cell (EC) lining and trigger platelet adhesion, leading to thrombosis and early occlusion. They also exhibit a mechanical mismatch with native vessels, causing high stress at suture lines [14,15]. Dacron grafts likewise achieve >85% five-year patency in the aorta yet fail in <6 mm vessels because of high stiffness and lack of elasticity, which cause intimal thickening at the anastomoses. Silicone-based elastomer grafts have also been tested, offering higher compliance, but have historically suffered from poor tissue integration and aneurysmal dilation in vivo [16]. Natural materials like collagen have been tested as grafts, but collagen is thrombogenic (promoting platelet adhesion and coagulation) and lacks sufficient mechanical strength and EC support [17,18,19]. Thus, no off-the-shelf graft adequately resists thrombosis, neointimal hyperplasia, and compliance mismatch in the small-caliber setting [6]. Recent reviews emphasize the need for biointegrated grafts that combine hemocompatibility with mechanical compliance to improve long-term patency. These limitations motivated us to design a plant-based scaffold capable of sustaining physiological loads in vivo.

Decellularization removes cellular components from tissues to produce acellular scaffolds. It has been widely applied to animal tissues to preserve native structural and mechanical properties. More recently, plants have been decellularized to create perfusable scaffolds, inspired by the analogous branching architecture of plant vasculature. Unlike animal tissue, plant cell walls are primarily composed of cellulose, a biocompatible polymer that promotes cell attachment and wound healing. Decellularized leaves can be repopulated with various mammalian cell types, including ECs, vascular smooth muscle cells (vSMCs), cardiomyocytes, osteoblasts, and human induced pluripotent stem cells [20,21,22]. Cevik et al. developed small-diameter vascular grafts from decellularized parsley stems. Prior studies, including our own, have fabricated plant-derived small-caliber tubular grafts and demonstrated endothelialization in vitro. However, comparative evaluation of multiple plant leaves for vascular applications revealed that decellularized leatherleaf viburnum exhibited superior mechanical integrity, more complete decellularization, and substantially higher burst pressures when fabricated into small-diameter grafts compared to spinach and parsley leaves [23]. Spinach- and parsley-based scaffolds showed reduced mechanical strength and were unable to reliably form robust 3D conduits, limiting their suitability for arterial implantation. To date, multilayer plant-based conduits have not yet been implanted for small-diameter arterial repair in vivo.

In this study, we engineered a multilayer, small-caliber vascular graft reinforced with gelatin using decellularized leatherleaf viburnum seeded with ECs and vSMCs, and tested its performance in vivo. The decellularization protocol used 2% sodium dodecyl sulfate (SDS) followed by bleach/Triton X-100 to completely remove plant cells and DNA while preserving the integrity of the ECM. To mimic a native three-layer vessel, we seeded the decellularized leaf with vSMCs, wrapped it around a temporary mandrel, and cross-linked it with gelatin to form a tubular scaffold. ECs were then perfused through the lumen, generating a fully endothelialized graft with mechanical properties and permeability comparable to native vessels. Once recellularized, the grafts were conditioned in a custom bioreactor that applied hemodynamic forces mimicking the in vivo microenvironment. We previously demonstrated that this preconditioning increases luminal EC density and reduces thrombosis in vitro, and that the grafts meet surgical standards for suture retention with high cell viability [24]. Next, we evaluated the in vivo performance of our bioreactor-conditioned plant-based grafts in a rat abdominal aorta interposition model, focusing on graft endothelialization, patency, and remodeling over time. Our approach utilizes the native plant ECM architecture combined with a multilayer cell-seeded design to enhance cell integration. To our knowledge, this is the first in vivo evaluation of a vascular graft derived from a decellularized plant scaffold. We hypothesized that the decellularized plant matrix would provide a conducive microenvironment for initial EC and vSMC adhesion and maintain vessel patency and that flow preconditioning would enhance long-term endothelialization and minimize early graft occlusions. This approach could offer an alternative to traditional synthetic and animal-derived grafts, introducing a potential new class of plant-based vascular grafts.

## 2. Materials and Methods

### 2.1. Plant Leaf Decellularization

Fresh leatherleaf viburnum from a single location was decellularized in 2% SDS (Sigma-Aldrich, St. Louis, MO, USA) for 72 h, followed by 6 h in a clearing solution (10% bleach and 0.1% Triton X-100) and sterilized for 10 min in 70% ethanol as previously described [23].

### 2.2. DNA Quantification

Intact and decellularized leatherleaf viburnum samples (30–45 mg per sample) were weighed and homogenized (MP PastPrep-24) using Lysing Matrix A (MP Biomedical). The samples (*n* = 3) were then digested with lysis buffer for 30 min at 56 °C. DNA was measured photometrically at 260 nm, as previously described [25].

### 2.3. SDS Quantification

Residual SDS in decellularized leatherleaf scaffolds was quantified using a Stains-All colorimetric assay (1-Ethyl-2-[3-(1-ethylnaphtho [1,2-d]thiazolin-2-ylidene)-2-methylpropenyl]naphtho [1,2-d]thiazolium bromide, Sigma-Aldrich) adapted from Rusconi et al. [26]. Decellularized leatherleaf samples were weighed, homogenized using Lysing Matrix A, extracted centrifuged to remove debris, and the supernatant was used for SDS quantification. Four samples were analyzed for SDS content using the Stains-All colorimetric assay. Samples were quantified using a standard curve prepared from serial dilutions ranging from 0 to 2% (*w*/*v*) SDS at an absorbance of 438 nm. The limit of quantification (LOQ), defined as the lowest concentration that can be reliably quantified with acceptable precision and accuracy, was 0.018 µg SDS per mg dry tissue.

### 2.4. Tensile Testing

Dog-bone specimens were cut from decellularized leatherleaf as previously described [27]. Gauge thickness, width, and length were measured for each specimen. Uniaxial tension was applied at 0.08 mm/s to failure. Maximum tensile load and elastic modulus were obtained from the load–extension curves after converting to stress and strain using the measured gauge dimensions.

### 2.5. Cell Culture

Primary rat vSMCs and rat aortic ECs were obtained from Cell Applications (San Diego, CA, USA) at passage three and passage two, respectively. The cells were expanded under standard incubator conditions and used through passages eight and sixteen, respectively. vSMCs were maintained in Smooth Muscle Cell Growth Medium (Cell Applications) and transitioned to Smooth Muscle Cell Induction Medium (Cell Applications) for differentiation. ECs were cultured in Rat Endothelial Cell Growth Medium (Cell Applications).

### 2.6. Graft Fabrication

Decellularized leatherleaf sheets were trimmed to 21 × 25 mm and functionalized with fibronectin (20 μg/mL). vSMCs were seeded on the abaxial surface at a density of 15,000 cells/cm^2^ and cultured at 37 °C for 24 h to promote initial attachment. Following recellularization, samples were formed into tubular constructs by wrapping the leaf around a 1.5 mm diameter stainless-steel mandrel, with the adaxial surface oriented toward the lumen (Supplemental Figure S1). To stabilize the construct, a gelatin-based adhesive was prepared by combining 50% bovine gelatin (Sigma–Aldrich) heated to 55 °C, with 25% glutaraldehyde (Sigma–Aldrich) and applied along one edge of the leaf prior to wrapping. Constructs were held in place for 30 s to permit cross-linking. Following cross-linking, grafts were gently rinsed in cell culture media to remove residual cross-linking reagents prior to incubation in media at 37 °C for 24 h. For luminal endothelialization, grafts were filled with fibronectin solution (20 μg/mL) using Luer Lock fittings and incubated for 1 h. ECs were subsequently introduced into the lumen at a density of 946,000 cells/cm^2^. Seeded grafts were maintained at 37 °C and rotated by 90° at 15 min intervals for 3 h to promote uniform cell distribution. As controls, silicone grafts were coated with fibronectin and seeded with ECs as described above, and additional grafts were constructed using decellularized leatherleaf viburnum without seeding of the vSMCs and then seeded with ECs.

After observing premature graft failure of plant-based grafts in the week 1 group, 44 μL of additional cross-linked gelatin was applied (after wrapping the scaffold around the mandrel) to the edges of grafts used in the 4-week and 24-week study to prevent bleeding after implantation.

### 2.7. Bioreactor Conditioning of Grafts

Using a custom-built bioreactor previously described, seeded grafts were preconditioned with 5 mL/min fluid flow at 37 °C [24]. Flow was gradually increased from 0.2 mL/min to 5 mL/min over 4 h to allow the cells to adapt to the increasing shear forces, after which grafts were maintained under continuous perfusion at 5 mL/min for 24 h. Perfusion culture was conducted using a peristaltic pump (Masterflex L/S, Cole-Parmer) connected to C-Flex tubing (L/S 15) and a custom-fabricated clear acrylic culture chamber (11.4 × 11.4 × 11.4 cm). The chamber walls were fitted with threaded ports accommodating NPT male single-barb connectors on the exterior and polypropylene Luer-lock fittings internally to allow graft cannulation. Prior to assembly, all chamber components and tubing were disinfected with 70% ethanol. The system was assembled under sterile conditions in a biosafety cabinet, treated with 10% bleach for 10 min, and rinsed thoroughly with deionized water followed by sterile PBS. The chamber was then filled with culture medium consisting of Medium 199 (Sigma-Aldrich) supplemented with 2% fetal bovine serum (BioTC, Wayne, NJ, USA) and 1× penicillin–streptomycin, and the tubing circuit was primed with media. Recellularized grafts were cannulated using 18-gauge needles, secured with elastic ties, and mounted within the chamber by coupling to the internal Luer-lock barbs. Wall shear stress within the graft lumen was estimated assuming laminar flow using the Poiseuille relationship, $\tau =\frac{4Qµ}{\pi r^{3}}$ where Q is the volumetric flow rate, µ is the dynamic viscosity of the culture medium, and r is the graft inner radius. For media viscosity µ of 6.92 × 10^−4^ N·s/m^2^, a flow rate of 5 mL/min generated a mean wall shear stress of 0.174 Pa within the 1.5 mm inner diameter vascular grafts. Although this shear stress is below typical arterial values, low shear preconditioning is commonly used to promote EC adhesion and retention on vascular graft surfaces before exposure to higher physiological stresses [28,29]. In prior graft-conditioning studies, EC-seeded conduits were first exposed to 1–2 dyn/cm^2^ to improve adherence and viability prior to being subjected to physiological shear (15–25 dyn/cm^2^). Consistent with this rationale, we previously found that shear stresses above 4 dyn/cm^2^ caused rapid EC detachment and subsequent cell death in vitro, supporting the use of a lower initial shear level for newly seeded constructs. Additionally, endothelial morphological adaptation and alignment occur over the first 12–24 h of shear exposure, supporting a 24 h conditioning period for initiating shear-responsive remodeling while maintaining cell retention [30,31].

### 2.8. Burst Pressure and Compliance Testing

Cell-seeded, bioreactor-conditioned plant-based grafts were mounted on barbed adapters. One end was connected to a pressure transducer, the other to a syringe pump. Specimens were pressurized with water at a constant rate while intraluminal pressure was recorded; the peak pressure immediately before rupture was taken as burst pressure, as previously described [25]. Compliance was evaluated per ANSI/ISO 7198:2016 from pressure–diameter data over 80–120 mmHg and reported as % per 100 mmHg [32].

### 2.9. Suture Retention Testing

Suture retention strength was measured on plant-based vascular grafts (*n* = 3), as previously described [24]. A single 10-0 Prolene stitch was placed 2 mm from the graft edge and tied to a custom suture holder; the graft was clamped in the opposing grip of a uniaxial tester with a 100 N load cell. The suture was pulled at 60 mm/min to failure, and the peak load was recorded as the suture retention strength. This pull rate and configuration follow recommended practice for vascular prostheses testing.

### 2.10. Permeability Testing

Permeability of plant-based grafts seeded with vSMCs and ECs and plant-based grafts seeded with only ECs was compared by perfusing food coloring at a pressure of 15.8 kPa (118 mmHg, physiological pressure) for up to 24 h in vitro. Twenty drops of blue food coloring were diluted in 20 mL of water and then perfused into each graft using a 10 mL syringe (Supplemental Figure S2). At the other end, a sensor was used to measure the resulting pressures. Pressure was maintained by connecting two rubber bands to the syringe plunger. The experiment was conducted in a bin of water to hydrate the grafts and better visualize when leakage occurred.

Permeability of 0.5 × 0.5 cm decellularized leatherleaf sheets was also measured for 120 min under static conditions (*n* = 3), as previously described [33]. Before testing, each sheet was seeded with ECs on one surface and vSMCs on the opposite surface to assess permeability of the seeded scaffold. Flux across the scaffold was recorded and normalized to area and time, and values are reported as mL/cm^2^/min in line with ISO 7198 guidance for vascular prostheses [32].

### 2.11. Animal Model Selection

Rats were chosen as the small animal model given that they are suitable in size and anatomy for arterial interposition grafts and have been used extensively in the previous literature for these types of experiments [34,35]. This size allows evaluation of vascular grafts of comparable size to human coronary arteries. Larger mammals are better for late-stage graft studies, but rats suffice for early characterization of complex interaction between blood, flow, and their reaction to the graft.

### 2.12. Surgical Approach

To prepare for graft implantation, we focused on optimizing graft diameter, suture size, and anastomotic technique. Grafts 1–2 mm in diameter were tested, and a 1.5 mm diameter minimized graft-to-artery size mismatch. We evaluated 7-0 to 10-0 Prolene sutures and found all of them suitable for visualization and handling, with a 6.5 mm, 3/8-circle micro-point needle providing the best anastomotic performance. In this study, continuous suturing resulted in superior anastomosis and minimized postoperative anastomotic leakage compared to interrupted sutures.

### 2.13. Study Design

For graft implantation, 8-week-old male Sprague-Dawley rats (251–275 g) were purchased from Charles River Laboratories and were free of visible illness. A total of 24 rats were used in this study. The study was conducted in a staged manner to evaluate both early feasibility and longer-term graft performance.

In the initial phase (1-week endpoint), animals were randomized to receive plant-based grafts seeded with ECs alone, plant-based grafts seeded with both vSMCs and ECs, or silicone grafts seeded with ECs (*n* = 3 per group). This early time point was designed to assess acute thrombosis, patency, and surgical feasibility. Following observation of consistent early thrombosis and non-survival in grafts lacking vSMCs, this group was not pursued at later time points, as reported in Section 3.4 (Surgical Outcomes).

In the subsequent phase, longer-term studies were conducted at 4 and 24 weeks to compare plant-based grafts seeded with vSMCs and ECs to silicone controls (*n* = 3 per group per time point). For these later studies, grafts were reinforced with additional gelatin to improve mechanical sealing and reduce postoperative bleeding, based on observations from the initial cohort.

Animals were euthanized if they exhibit predefined clinical symptoms associated with non-survival or illness. Specifically, animals were euthanized if they had signs of dyspnea; impending respiratory or cardiac arrest, such as agonal breathing; bradypnea (respiratory rate of 30 per minute or less); if heart rate is below 150 beats per minute; greater than 20% weight loss or a body temperature below 34 °C. These animals would be defined as non-survivors. In addition to the previous symptoms, signs of illness may also include an inability to access food or water, pain, swelling, redness or discharge from surgical incisions.

Silicone grafts were selected as the control because they are biocompatible, flexible, and transparent, which facilitated surgical handling and visualization of blood flow during and after implantation [36,37]. Silicone has also been reported to exhibit reduced calcification and inflammation compared to ePTFE and polyurethane, and its smooth surface helps minimize clot formation at smaller diameters.

### 2.14. Vascular Graft Implantation

Rats were housed individually in ventilated cages with animal bedding in an animal facility under a 12 h light/dark cycle maintained at 21 ± 2 °C and 40–60% humidity. Animals fasted 12 h before surgery.

The rats were anesthetized with 4% isoflurane/100% oxygen in an induction chamber for 3–4 min. After induction, anesthesia was maintained at 1–2% isoflurane in 100% O_2_ (flow rate 400–1000 mL/min) for 10 min. The isoflurane level was adjusted for each animal until no response to a toe pinch was observed. A mask breathing circuit and chemical scavenger were used (Supplemental Figure S3). The surgical site was prepared with povidone-iodine and alcohol, and then the midline of the abdomen was cut longitudinally to expose abdominal cavity. The abdominal aorta was exposed and dissected, clamped and transected at 10 mm below the bifurcation of the renal artery. A measure of 100 U heparin was injected into the aorta before clamping to prevent distal thrombosis. A 10 mm segment of abdominal aorta artery was removed and the recellularized graft (10 mm long) was interpositioned by end-to-end anastomosis using continuous 10-0 polypropylene (Prolene) monofilament sutures. A biocompatible gelatin-based sealant was applied to prevent postoperative anastomotic leakage while promoting tissue repair. After application of the gelatin-based sealant, sterile gauze was briefly applied to the graft to wick away excess gelatin prior to closure. Sutures were used to close the incision.

After each operation, ketoprofen (5 mg/kg) was administered to the animals. A warming blanket and subcutaneous fluids were used during a period of post-operation intensive care to prevent hypothermia and/or dehydration. A pulse oximeter was used to determine oxygen saturation of arterial blood and heart rate. No antiplatelet treatment was used post-operatively.

### 2.15. Vascular Graft Ultrasonography

Initial assessment of patency for the plant-based and silicone vascular grafts, in comparison with the native vessel (control), was performed using several physiological indicators: vessel diameter, flow rate, systolic and diastolic velocities, resistive index (RI), pulsatility index (PI), and a novel image-based heterogeneity index (HI) [38]. Then, we confirmed that the last three would be the optimal markers for assessing patency. These index comparisons are based on mean values of pooled data across 1-week, 4-week, and 24-week time points for both the plant-based and the silicone grafts, as compared to a single control case. One related point is that the number of data points collected was small for the control and for the plant-based and silicone grafts. In addition, the pooled data was not (in most cases) longitudinal. As a result, we were unable to determine in this portion of the study whether any of the aforementioned comparisons showed statistically significant differences.

RI and PI quantify arterial resistance on Doppler ultrasound. They are defined by:

$$RI=\frac{{SV}_{p}-{DV}_{e}}{{SV}_{p}}\;\;\;\;PI=\frac{{SV}_{p}-{DV}_{e}}{{SVDV}_{m}}$$

where ${SV}_{p}$ is peak systolic velocity, ${DV}_{e}$ is the end diastolic velocity and ${SVDV}_{m}$ is the mean velocity.

Flow rates, systolic and diastolic velocities, RI, and PI within the grafts were measured using Doppler imaging (Supplemental Figure S4B). The flow rates, Q, were calculated using:

$$Q={SVDV}_{m}\times A_{v}$$

where $A_{v}$ is the cross-sectional area ($\pi r_{v}^{2}$) of the vessel, and $r_{v}$ is the radius of the vessel.

Grayscale vascular images were obtained with a transverse view of the implantation site along the abdominal aorta (Supplemental Figure S4A). Occasionally, to confirm the proper location of the implant site post-surgery, a small reflector was placed beneath it as indicated by the hyperechoic feature in the figure. Using a GE LOGIQ e ultrasound system with a GE L8-18i multi-frequency probe and ultrasound transmission gel placed proximal to the implantation site, tests were performed in vascular mode. The images were digitally collected in the original Digital Imaging and Communications in Medicine (DICOM) format, which were converted to JPEG format and analyzed using MATLAB version 9.12.01884302 R2022a (The MathWorks, Inc, Natick, MA, USA).

Tissue heterogeneity in the implant and in the native vessel was determined from the ultrasound B-scan images in the area adjacent to the vessel. This quantification (HI) was based on dynamic range calculations using Floyd-Steinberg’s dithering technique. This method transforms the pixels in the image into a binary map [39,40]. A region of interest (ROI) was manually selected in the area adjacent to the vessel wall, excluding areas with shadowing (Supplemental Figure S4C). The ROI is further divided into sub-ROIs with 100 pixels in each. The algorithm assigns each pixel in the ROI/sub-ROI to either a 0 (black) if its intensity falls below 50% of the grayscale or a 1 (white) if its intensity is above 50%. It is assumed that the grayscale values are in the intensity range between 0 and 255. The dithered binary map version of the image is generated within the ROI (Supplemental Figure S4D). The HI value for each dithered image can then be calculated by first storing the percentages of the number of white pixels to the total number of pixels in each of the sub-ROIs. Then, the average of the five highest and the five lowest percentages in the whole ROI are determined. Finally, the ratio of the two averages is the HI value for the image.

### 2.16. Histology and Staining

At 1, 4, and 24 weeks, explanted grafts were sectioned from the middle part and fixed in 4% formaldehyde for 1 h and sent to VitroVivo Biotech (Rockville, MD, USA) for paraffin embedding (Supplemental Figure S5). The mid-graft region was selected for histology to enable consistent assessment of graft body remodeling while minimizing confounding effects from suture lines and anastomotic hemodynamics, which are known to promote localized thrombus formation and neointimal hyperplasia. Complementary SEM analysis was also used to evaluate surface features at the proximal and distal graft ends, where edge-associated thrombus is expected to be most prominent. These histological samples were stained with hematoxylin and eosin (H&E), Picro Sirius Red, and Verhoeff-van Gieson to examine cell presence and infiltration, collagen, and elastin, respectively. Brightfield microscopy was performed at 40× magnification using an Olympus FV3000 confocal microscope (Olympus Corporation, Tokyo, Japan). The luminal wall area and wall thickness were measured for grafts and native aorta using H&E images in ImageJ (version 1.54r) for 3 images per sample (*n* = 9). Collagen coverage and elastin coverage were calculated using 3 images per sample stained with Picro Sirius Red and Verhoeff-van Gieson (*n* = 9). Assessors for histology were blinded to group allocation. Tissue sections were also stained for Hoechst nuclear counterstain to assess EC coverage. Blinded evaluators manually counted nuclei in three confluent regions on each sample (*n* = 9) using fluorescent microscopy at 40× magnification.

### 2.17. Scanning Electron Microscopy

The morphology of the explanted grafts at 1, 4, and 24 weeks was also examined by scanning electron microscopy (SEM) and compared to grafts prior to implantation to determine if any tissue remodeling occurred. Samples were fixed with 2.5% glutaraldehyde. Following fixation, samples were dehydrated through a graded ethanol series (70–100%) as previously described [23]. Specimens were then dried using a critical point dryer (Samdri-795, Tousimis, Rockville, MD, USA), mounted on aluminum stubs, and sputter-coated with gold (EMS-550). Imaging was performed using a Quanta FEI-250 scanning electron microscope at 200× and 800× magnification.

These images were converted to 8-bit, then contrast and entropy were measured using the GLCM Texture Analyzer, a plugin of ImageJ. Next, these 8-bit images were binarized, and fractal dimensions were calculated using the box counting method in FracLac, a plugin of ImageJ, as previously described [41]. Additionally, thrombus surface area fraction (%) was quantified from binarized SEM images as the percentage of the luminal surface occupied by thrombus, and thrombus thickness (µm) was measured from SEM images as the maximum width of thrombus deposits along the luminal surface. For each test, 3 images per sample (*n* = 9) were used. Evaluators performing SEM image analysis were blinded to group allocation.

### 2.18. Collagen and Elastin Content

The collagen and elastin contents of the explanted grafts and native aorta artery were tested using standard assay kits and compared to control grafts to quantify ECM deposition at 1, 4, and 24 weeks after implantation. The collagen content was measured with a hydroxyproline assay and based on a quantitative colorimetric determination and compared to a collagen standard (QuickZyme Biosciences). Elastin content was measured using a Fastin Assay Kit utilizing a dye-based method to quantify various elastin forms with colorimetric detection (Biocolor Ltd., Belfast, UK). Collagen and elastin content were normalized by the weight of each sample and we compared the measured amounts in each group with those of native rat aorta.

### 2.19. Statistical Analysis

The sample size for the number of animals used in this study was calculated with G*Power (version 3.1.9.7) [42]. The required sample size for 80% power, α = 0.05 type I error, two tails, and f = 0.60 effect size was calculated as 24. An effect size between 0.6 and 0.7 has been reported for animal cardiac surgery [43,44]. An Anderson-Darling test was used to assess the normality of the data collected in this study. Comparisons among three or more groups were made using one-way analysis of variance (ANOVA). When a significant main effect was detected, Tukey’s post hoc test was applied to assess pairwise differences. Statistical significance was defined as *p* < 0.05, and analyses were conducted through Microsoft Excel (Redmond, WA, USA). For comparisons between two groups, independent two-tailed *t*-tests were performed with a significance threshold of *p* < 0.05. Data are expressed as mean ± standard deviation. All hypothesis tests were two-tailed with α = 0.05.

## 3. Results

### 3.1. Characterization of Decellularized Leatherleaf

**Figure 1:**
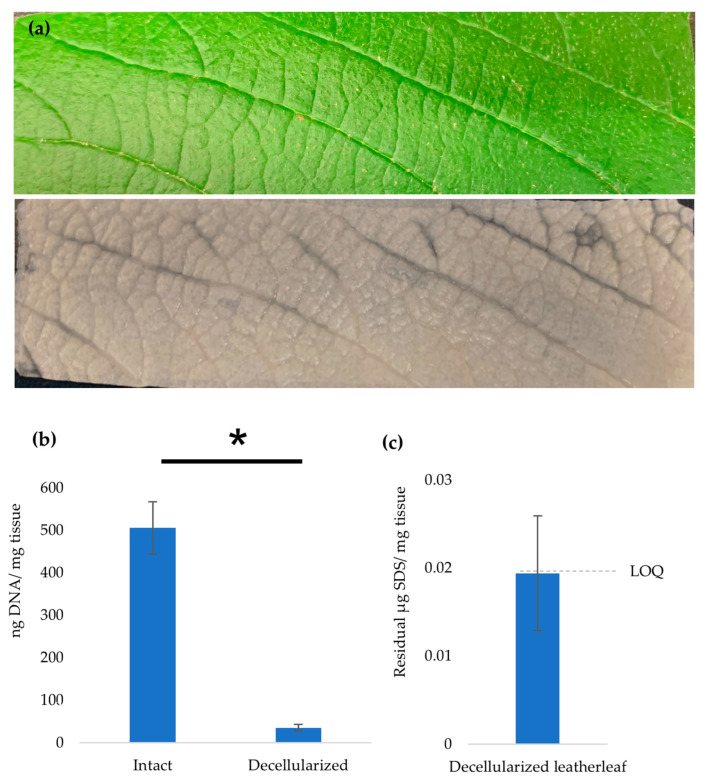
(**a**) Images of leatherleaf viburnum before and after decellularization showing removal of cellular material. (**b**) DNA content of native and decellularized leatherleaf showing effective removal of DNA following decellularization. (**c**) Residual SDS content in decellularized leatherleaf measured using the Stains-All assay. * *p* < 0.05 and error bars represent standard deviation. The limit of quantification for SDS measurement was 0.018 µg SDS/mg tissue.

We assessed the pre-implant state of the scaffolds. The leatherleaf viburnum scaffolds were whitish and opaque following decellularization with SDS and clearing solution (Figure 1a). DNA quantification confirmed cell removal, with DNA contents of 506 ± 61 and 35 ± 8 ng DNA/mg tissue for intact and decellularized leatherleaf, respectively (Figure 1b). The Stains-All standards exhibited a graded color change with increasing SDS, consistent with prior descriptions of the assay’s response. Residual SDS values were near the LOQ (0.018 µg SDS/mg tissue), indicating minimal detergent remaining in the scaffold (Figure 1c. DNA quantification is widely used as a primary metric to validate effective decellularization, with reduced DNA content indicating successful removal of cellular material while preserving ECM structure. This approach is consistent with established decellularization validation strategies that combine biochemical DNA assessment with histological evaluation in decellularized biomaterial scaffolds [45].

### 3.2. Mechanical Performance of Scaffolds and Grafts

We evaluated mechanical performance of the decellularized leatherleaf viburnum scaffolds and the plant-based vascular grafts in vitro (Table 1). Dog-bone strips cut from decellularized leatherleaf showed a maximum tensile load of 0.32 ± 0.09 N and an elastic modulus of 2.98 ± 0.92 MPa (*n* = 3). For the tubular grafts, 10-0 suture retention strength was 0.85 ± 0.11 N (*n* = 3). Pressurization of the grafts yielded a burst pressure of 409.8 ± 43.3 mmHg, and graft compliance calculated from the pressure–diameter relationship over 80–120 mmHg was 3.41 ± 0.70% per 100 mmHg (*n* = 3).

### 3.3. Vascular Permeability In Vitro

In vitro perfusion testing demonstrated that grafts seeded with both ECs and vSMCs could withstand >15.8 kPa for 24 h (physiological pressure) without leakage, whereas grafts seeded with ECs alone began leaking at 10.3 kPa. This suggests that vSMCs provide additional support, allowing the graft to maintain its integrity under higher pressure. Permeability was also evaluated under static conditions using leatherleaf viburnum scaffolds seeded with ECs on one surface and vSMCs on the opposite surface; no leakage was observed over 2 h (*n* = 3; Supplemental Figure S3B).

### 3.4. Surgical Outcomes

At the 1-week time point (n = 12), 33% of rats receiving plant-based grafts seeded with vSMCs and ECs (1 of 3) survived, and none with ECs alone (0 of 3) survived the whole week. After observing 100% early thrombosis in grafts without vSMCs at 1 week, we did not pursue this group at longer time points. Among rats with silicone grafts, 17% (1 of 6) survived to 1 week. Non-survivors often exhibited hind-limb paralysis by day 2 and internal bleeding at necropsy.

For the 4-week time point (*n* = 6), grafts were reinforced with additional gelatin to improve mechanical sealing and reduce postoperative bleeding. All rats with the reinforced plant-based grafts survived 4 weeks (3 of 3, 100%), whereas 67% of those with silicone grafts survived (2 of 3). One rat with a silicone graft died in the first week. Autopsies revealed scar tissue around the graft sites in all rats, but no acute failures of the plant-based grafts were observed.

**Figure 2:**
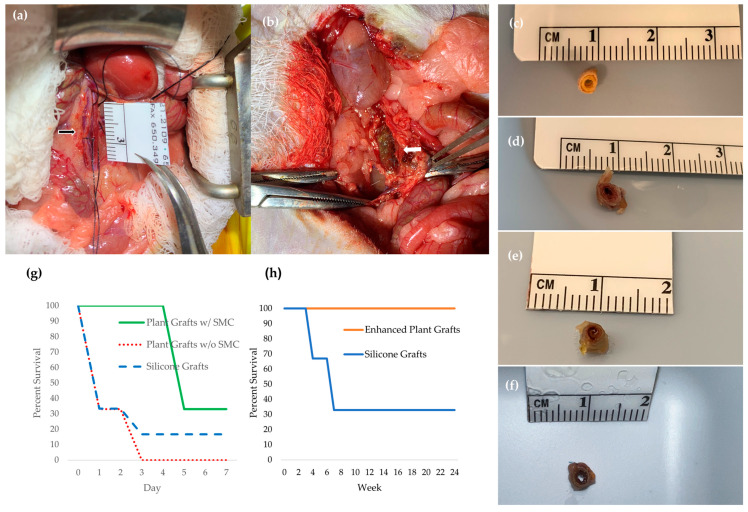
(**a**) Aortic exposure followed by (**b**) implantation of plant-based graft by end-to-end anastomosis in rat. Photos of cross-section of plant-based graft (**c**) before implantation and explanted (**d**) 1, (**e**) 4, and (**f**) 24 weeks after implantation. Percent survival for rats implanted with plant-based grafts and silicone grafts for (**g**) 1 week and (**h**) 4–24 weeks. The black arrow indicates the native aorta, and the white arrow indicates the implanted graft.

In the 24-week cohort (*n* = 6), 100% of rats with reinforced plant-based grafts survived to 24 weeks. One was euthanized at 6 weeks due to a complication (a kidney embolism) unrelated to graft integrity and was excluded from the analysis. The graft itself remained patent and intact. In contrast, only 33% of rats with silicone grafts survived to 24 weeks (1 of 3); the other two died by weeks 4 and 6. Autopsies in the silicone group noted an enlarged kidney in one case and abdominal bloating in another, possibly due to secondary vascular issues, along with scar tissue near all grafts. Kaplan–Meier curves summarize survival for each group (Figure 2g,h). Figure 2g depicts the 1-week survival probability: plant vSMC+EC 33% (1/3), silicone 17% (1/6), plant EC-only 0% (0/3) by day 7. Figure 2h shows survival from 4 to 24 weeks: the plant graft group maintained a higher survival probability than the silicone group throughout the study period. All animals recovered from anesthesia.

### 3.5. Vascular Patency and Flow Rate In Vivo

**Figure 3:**
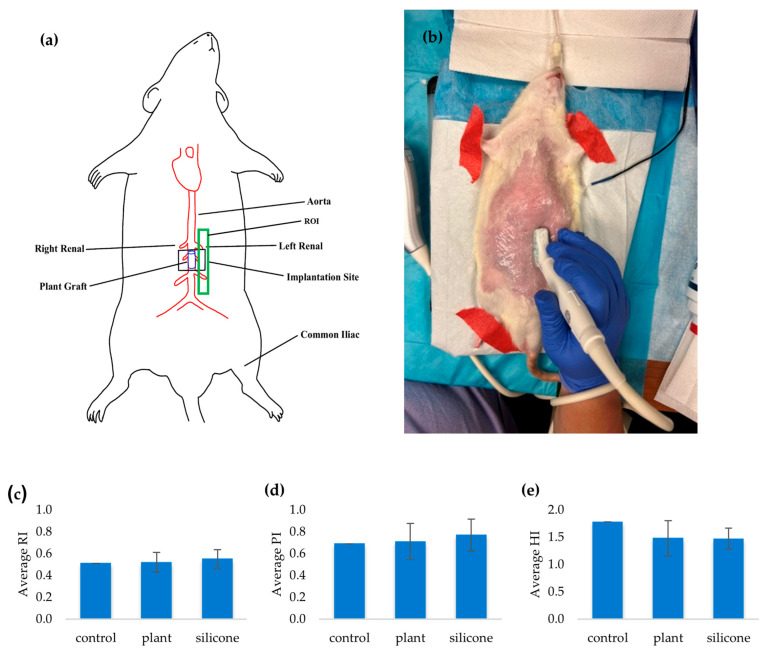
(**a**) Vascular graft ultrasonography showing the location of the graft (within the black box) and ROI (green box) adjacent to graft site where HI was measured, and (**b**) placement of ultrasound probe along the longitudinal direction of the vessel. Bar plots of (**c**) average RI, (**d**) average PI, and (**e**) average HI.

Several indicators were used to assess graft patency compared to the native aorta. Initially, patency, defined as the ability of a blood vessel to allow passage without obstruction, was evaluated by measuring vessel diameter, flow rate, and systolic and diastolic velocities. However, the most informative parameters were the RI and PI, which were derived from systolic and diastolic velocities and reflected vascular resistance and pulsatile flow. We also introduced a novel measure, the HI, to quantify changes in the tissue adjacent to the graft; its calculation is described in the Materials and Methods. RI, PI, and HI were assessed at 1, 4, and 24 weeks post-implantation using a GE LOGIQ e ultrasound system with an L8-18i multi-frequency probe. Tests were performed in vascular mode using Doppler imaging. Due to the limited sample size for Doppler-derived measurements, statistical comparisons between groups for RI, PI, and HI were not performed, and the reported values are presented for descriptive comparison only. The probe was positioned longitudinally along the vessel to obtain the strongest signal, then angled for optimal RI and PI measurements (Figure 3a,b).

Once grayscale B-mode images were acquired, HI values were measured within an operator-defined ROI adjacent to the graft site (green box in Figure 3a). Bar plots of average RI and PI (Figure 3c,d) suggest the plant-based grafts achieved near-normal flow characteristics (RI 0.52 vs. 0.51; PI 0.71 vs. 0.69), whereas silicone grafts showed higher resistance indices (RI 0.55; PI 0.77), indicating greater resistance which explains the rougher inner surfaces these grafts exhibited. HI values (Figure 3e) were lower in both graft groups (1.48 for plant-based and 1.47 for silicone) compared to native tissue (1.78), representing a 17.2% reduction. We attribute this to compression of adjacent tissue by the stiffer graft materials, resulting in a more homogeneous appearance. We hypothesize that native tissue, composed of adipose, connective, and muscle layers, is inherently heterogeneous. From a comparative standpoint, plant grafts differed from native vessels by only 1.96% in RI and 2.90% in PI, whereas silicone grafts differed by 7.84 and 11.59%, respectively. These findings suggest that the plant-based graft produces hemodynamics that more closely resemble those of the native vessel, supporting our conclusion that they remain patent.

### 3.6. Histological Analysis and Staining

**Figure 4:**
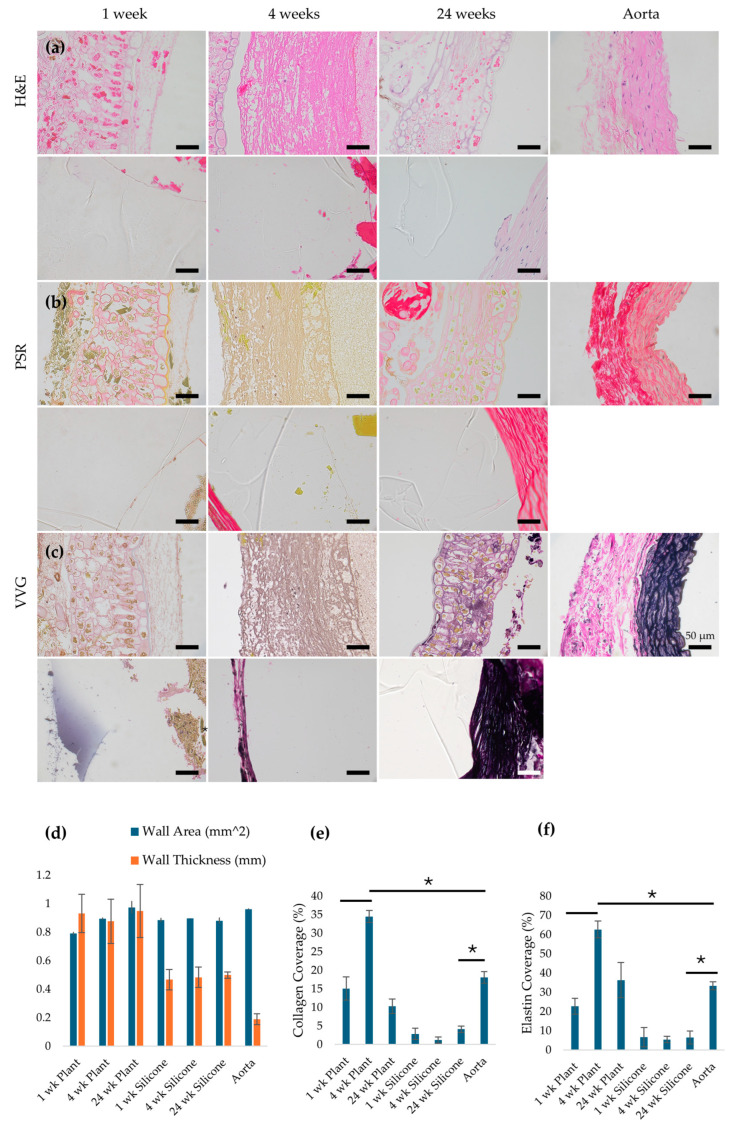
Images of histology for (**a**) H&E, (**b**) Picro Sirius Red, and (**c**) Verhoeff-van Gieson stains for plant-based grafts seeded with both vSMCs and ECs, silicone grafts, and native aorta 1, 4, and 24 weeks after implantation at 40× magnification. In H&E images, nuclei appear blue; Picro Sirius Red stains collagen red; and Verhoeff-van Gieson stains elastin black. The EC-only plant graft group is not shown beyond 1 week due to early thrombosis and non-survival. (**d**) Luminal wall area and wall thickness measured for grafts and native aorta using H&E images. (**e**) collagen coverage and (**f**) elastin coverage measured using images of Picro Sirius Red and Verhoeff-van Gieson stained grafts. * *p* < 0.05 and error bars represent standard deviation. One-way ANOVA and Tukey post hoc tests were used to compare data between groups with a *p* value of <0.05 to determine statistical significance.

Histological analysis was performed on plant-based grafts seeded with both vSMCs and ECs at all time points, as the EC-only graft group did not progress beyond the 1-week endpoint due to early thrombosis. H&E staining (Figure 4a) demonstrated that plant-based grafts maintained an open, circular lumen at 1, 4, and 24 weeks after implantation, with no evidence of luminal collapse or occlusive thrombus in surviving grafts. Histological examination (Figure 4a) showed that the luminal cross-sectional area of explanted plant-based grafts (0.79 ± 0.01, 0.90 ± 0.01, and 0.97 ± 0.04 mm^2^ at 1, 4, and 24 weeks) was comparable to that of native aorta (0.96 ± 0.01 mm^2^) and silicone grafts (0.88 ± 0.02 to 0.90 ± 0.01 mm^2^) (Figure 4d). However, the wall thickness of explanted plant-based grafts (0.93 ± 0.13, 0.88 ± 0.16, 0.95 ± 0.19 mm at 1, 4, 24 weeks) was significantly greater than that of silicone grafts 0.47 ± 0.07, 0.48 ± 0.07, and 0.50 ± 0.02 mm) and native aorta (0.19 ± 0.04 mm) at all time points (*p* < 0.05). This increased wall thickness reflects the presence of the plant-derived scaffold and associated tissue remodeling rather than pathological wall thickening. Collagen and elastin staining (Figure 4b,c) indicated that after 1 week, the plant grafts had collagen (red) and elastin (black) coverage comparable to that of the native aorta and higher than that of silicone grafts. At 4 weeks, collagen and elastin staining were more pronounced and broadly distributed within the graft wall, consistent with increased ECM deposition. Quantitative analysis confirmed that collagen and elastin coverage increased at 4 weeks and subsequently decreased by 24 weeks, suggesting a transition from early matrix deposition toward a more stabilized tissue structure.

**Figure 5:**
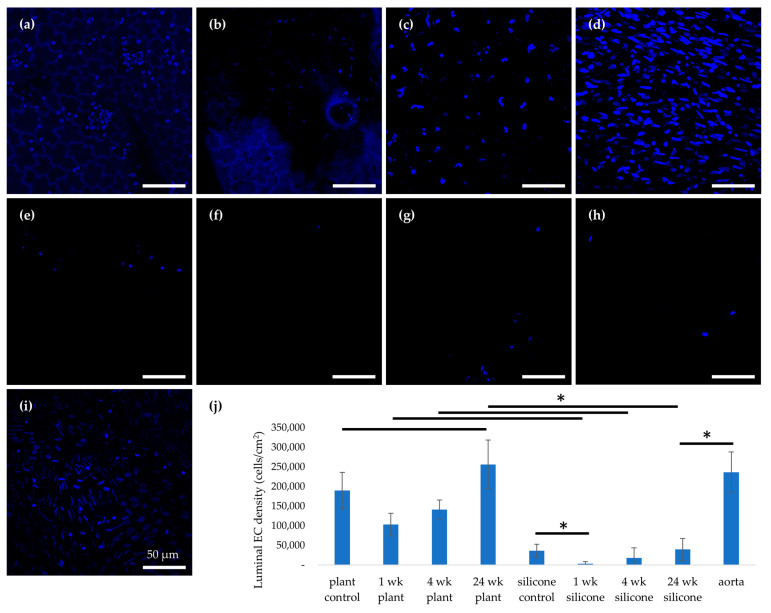
Representative 40× magnification images of endothelial cells on the lumen of (**a**–**d**) plant-based grafts and (**e**–**h**) silicone grafts before implantation, 1, 4, and 24 weeks after implantation, and (**i**) native aorta (*n* = 3), stained with Hoechst. Hoechst staining labels cell nuclei in blue. (**j**) Quantification of endothelial cell density for each condition. * *p* < 0.05 and error bars represent standard deviation. Cells were quantified from 3 fields per sample (*n* = 9). Statistical comparisons were performed using one-way ANOVA followed by Tukey’s post hoc tests, with significance defined as *p* < 0.05.

Hoechst nuclear staining (Figure 5) was used to quantify luminal EC coverage. Luminal endothelial coverage was quantified by counting Hoechst-stained nuclei, consistent with DNA- and nuclear-based quantification approaches commonly used to assess cell presence within regenerative biomaterial scaffolds [46,47]. Although plant-derived scaffolds can exhibit autofluorescence, Hoechst-stained nuclei of ECs can be reliably visualized and quantified in decellularized leatherleaf viburnum, as previously characterized [27]. Before implantation, the EC density on plant-based grafts (190,000 ± 46,043 cells/cm^2^) was comparable to that of the native aorta (236,666 ± 51,639 cells/cm^2^, *p* > 0.05) and significantly higher than on silicone grafts (36,666 ± 16,329 cells/cm^2^, *p* < 0.05). After 1 week in vivo, the plant grafts retained a higher EC density than silicone grafts (103,333 ± 28,751 vs. 3333 ± 5163 cells/cm^2^, *p* < 0.05), though both were lower than the native aorta. At 4 weeks, EC density on plant grafts (141,667 ± 24,013 cells/cm^2^) remained significantly greater than on silicone grafts (18,333 ± 25,625 cells/cm^2^, *p* < 0.05). By 24 weeks, the EC density on plant grafts (256,667 ± 61,860 cells/cm^2^) was statistically equivalent to that of the native aorta (236,667 ± 51,639 cells/cm^2^, *p* > 0.05) and much higher than on silicone grafts (40,000 ± 28,284 cells/cm^2^, *p* < 0.05). While the plant grafts experienced an initial drop in EC coverage by week 1, they showed a significant rebound by weeks 4 and 24, achieving a nearly native endothelial lining.

### 3.7. Extracellular Matrix Deposition and Tissue Remodeling

**Figure 6:**
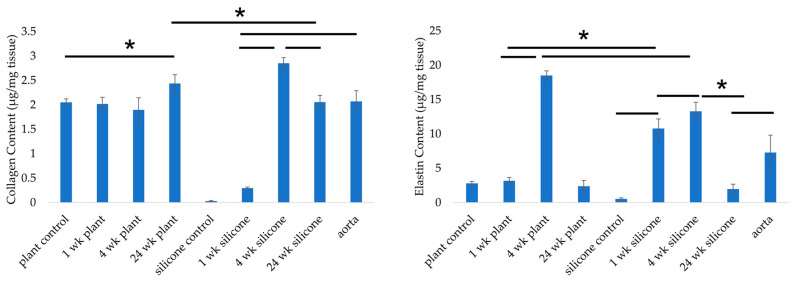
Collagen and elastin contents of grafts explanted at weeks 1, 4, and 24, as well as native vessel and control grafts. * *p* < 0.05; error bars indicate standard deviation (*n* = 6). Statistical significance was assessed using one-way ANOVA with Tukey’s post hoc test.

The collagen content in the plant-based grafts prior to implantation was 2.05 ± 0.07 µg/mg, comparable to that of the native aorta (*p* > 0.05) (Figure 6). At 1- and 4-week post-implantation, collagen content in the plant grafts did not change significantly (*p* > 0.05), but by 24 weeks, it had increased to 2.44 ± 0.18 µg/mg (*p* < 0.05). In the silicone grafts, total collagen increased significantly at 1 week (0.30 ± 0.02 µg/mg), 4 weeks (2.85 ± 0.11 µg/mg), and 24 weeks (2.05 ± 0.14 µg/mg) compared to unimplanted control grafts (0.03 ± 0.01 µg/mg). Collagen content at 1 week post-implantation was comparable to that of decellularized plant grafts prior to implantation, suggesting that the gelatin-based vascular sealant applied during surgery did not measurably contribute to the collagen signal.

The elastin content of the plant-based grafts prior to implantation was 2.80 ± 0.29 µg/mg, which was lower than that of the native aorta (*p* < 0.05). It did not change at 1 week (*p* > 0.05) but increased significantly by 4 weeks (*p* < 0.05) before decreasing by 24 weeks (*p* < 0.05). Elastin also significantly increased in silicone grafts at 1 week (10.79 ± 1.39 µg/mg) and 4 weeks (13.28 ± 1.32 µg/mg) compared to the control (0.53 ± 0.15 µg/mg), followed by a decline by week 24 (*p* < 0.05).

**Figure 7:**
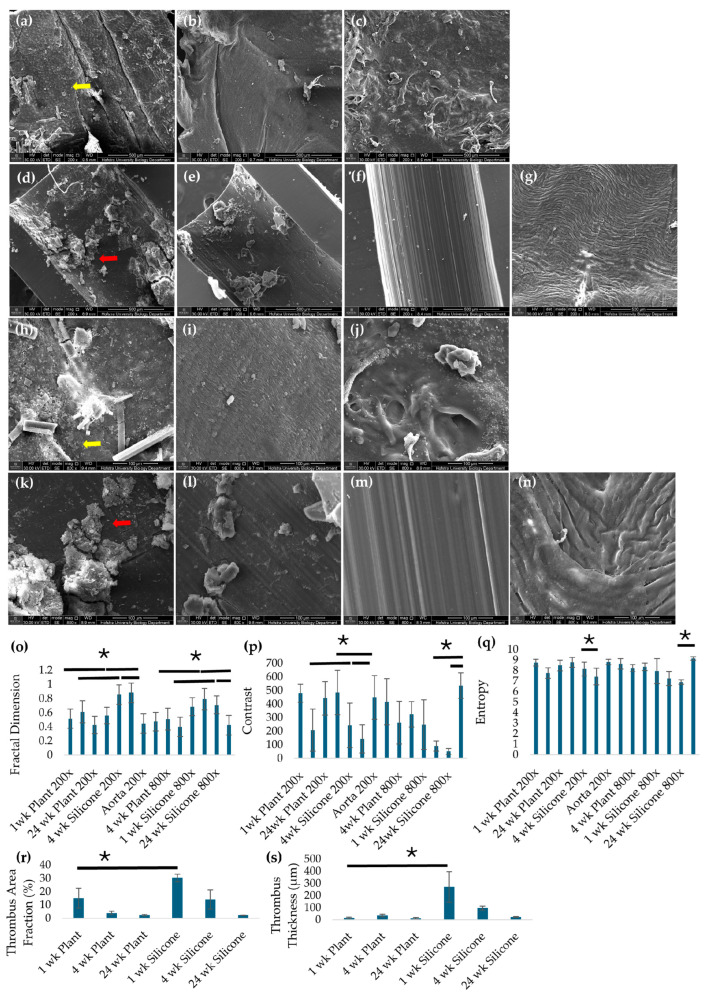
Representative SEM images of plant-based grafts seeded with both vSMCs and ECs at (**a**) 1, (**b**) 4, and (**c**) 24 weeks after implantation, silicone grafts (**d**) 1, (**e**) 4, and (**f**) 24 weeks after implantation, and (**g**) rat aorta imaged at 200× magnification, respectively. Representative SEM images of plant-based vSMC+EC grafts (**h**) 1, (**i**) 4, and (**j**) 24 weeks after implantation, silicone grafts (**k**) 1, (**l**) 4, and (**m**) 24 weeks after implantation, and (**n**) rat aorta imaged at 800x magnification. The EC-only plant graft group was not evaluated beyond 1 week due to early thrombosis and non-survival. Yellow arrows indicate endothelial cells on the lumen, and red arrows indicate thrombus formation. (**o**) Fractal dimension, (**p**) contrast, and (**q**) entropy measured from SEM images at 200× and 800× for plant-based grafts, silicone grafts and native aorta 1, 4, and 24 weeks after implantation. (**r**) Thrombus surface area fraction (%) and (**s**) thrombus thickness (µm) quantified from SEM images. * *p* < 0.05 and error bars represent standard deviation (*n* = 6). One-way ANOVA and post hoc Tukey tests were used to compare data between groups with a *p*-value of <0.05 to determine statistical significance.

SEM analysis of explanted grafts (Figure 7a–n) revealed that plant-based grafts seeded with both vSMCs and ECs exhibited relatively smooth luminal surfaces and resembled the native aorta at 1, 4, and 24 weeks, with minimal thrombus formation. The luminal surface of plant-based grafts appeared uniformly covered, with few regions of exposed underlying scaffold or discontinuity in the cellular layer. In contrast, silicone grafts exhibited significantly more thrombus formation at 1 and 4 weeks and a rougher inner surface. SEM images of silicone grafts frequently showed irregular surface topography, discontinuous coverage, and exposed graft substrate associated with adherent thrombotic material. Quantitative SEM image texture analysis supported these qualitative observations. At both 200× and 800× magnification, silicone grafts explanted at 4 and 24 weeks had a significantly higher fractal dimension and lower contrast and entropy (Figure 7o–q) compared to native aorta and plant grafts (*p* < 0.05). Plant-based grafts at all time points showed fractal dimension, contrast, and entropy values similar to native aorta (*p* > 0.05), indicating that their luminal surface complexity and roughness remained low and comparable to a normal vessel. Contrast and entropy measure local intensity variation to identify surface irregularities and reflect the randomness in texture, respectively, for evaluating complex or heterogeneous surfaces. Together, these metrics quantitatively reflect differences in surface continuity and thrombus-associated heterogeneity between graft types. Quantification of thrombus surface area fraction from SEM images confirmed these observations. At 1 week, silicone grafts exhibited a significantly higher thrombus area fraction (30.36 ± 2.98) compared to plant-based grafts (15.13 ± 7.49), while thrombus coverage on plant-based grafts remained low and decreased further at 4 and 24 weeks (3.95 ± 1.37 and 2.35 ± 0.53, respectively). Thrombus area fraction on silicone grafts decreased at later time points (14.18 ± 7.17 at 4 weeks and 2.23 ± 0.23 at 24 weeks), resulting in similar low thrombus coverage between groups by 24 weeks. Consistent with surface coverage measurements, thrombus width was substantially greater on silicone grafts at early time points, with a mean thrombus width of 271.40 ± 126.69 µm at 1 week compared to 16.26 ± 6.47 µm on plant-based grafts. Thrombus width on silicone grafts decreased at 4 and 24 weeks (96.72 ± 15.29 µm and 23.49 ± 3.90 µm, respectively), whereas plant-based grafts maintained low thrombus width across all time points (35.61 ± 11.75 µm at 4 weeks and 13.37 ± 5.43 µm at 24 weeks).

### 3.8. Structure–Function Summary

As summarized in Table 2, key structural and morphological differences between the graft types correspond with the observed functional outcomes. The plant-based grafts present a biomaterial matrix designed to support cellular integration and a comparatively smooth, continuous luminal surface with higher EC coverage over time, whereas silicone grafts exhibited a rougher, discontinuous blood-contacting surface with greater early thrombus burden. Because thrombosis and loss of patency in small-diameter grafts are strongly influenced by the blood-contacting surface and the extent of endothelialization, these paired observations provide a clear structure–function framework for interpreting the in vivo results: grafts with a more confluent EC lining and smoother luminal morphology are associated with reduced thrombogenicity and improved patency trends in this study.

## 4. Discussion

In this study, our bioreactor-conditioned plant-based vascular grafts exhibited encouraging in vivo remodeling and patency compared to silicone grafts through 24 weeks. The results supported our hypothesis that the microenvironment provided by the decellularized plant tissue would promote initial EC and vSMC adhesion and maintain vessel patency and that the addition of fluid shear stress preconditioning would promote long-term endothelialization and minimize early graft occlusions in vivo. The plant-based grafts supported the formation of a more robust and continuous endothelial layer, as confirmed by nuclear counterstaining and SEM of the luminal surface. Survival at 4 weeks and 24 weeks was 100%, compared to 67% and 33% for silicone grafts, respectively. Among survivors, Doppler confirmed patency in all plant-based grafts. SEM imaging of plant-based grafts explanted at 1, 4, and 24 weeks revealed a smooth luminal surface, similar to that of native aorta, and a rougher luminal surface on implanted silicone grafts. Collagen and elastin content and coverage in the explanted plant-based grafts were comparable to those of the native aorta at week 1, but elastin increased significantly by week 4. This marks the first time that plant-based vascular grafts have been evaluated in vivo.

Decellularized leatherleaf scaffolds exhibited a maximum tensile load of 0.32 ± 0.09 N and an elastic modulus of 2.98 ± 0.92 MPa, indicating a compliant sheet suitable for integration with the graft construct. The plant-based vascular grafts withstood a 10-0 suture retention load of 0.85 ± 0.11 N, which supports handling at the anastomosis but requires careful technique. Pressure testing yielded a burst pressure of 409.8 ± 43.3 mmHg, approximately 3.4 times systolic pressure, providing an adequate safety margin. Compliance between 80 and 120 mmHg was 3.41 ± 0.70% per 100 mmHg, intermediate between native small arteries and stiffer synthetics, which may lessen but not eliminate the risk of compliance-mismatch–driven intimal hyperplasia. No leakage was detected under the test conditions. Together with pre-implant characterization demonstrating effective decellularization and low residual detergent, the mechanical profile supported further in vivo evaluation of these plant-based grafts.

Endothelialization of our plant-based grafts before implantation was comparable to that of the native aorta, and 24 weeks after implantation, EC density was statistically equivalent to that of the native aorta and significantly higher than that of silicone grafts, suggesting durable endothelial retention. This outcome supports the hypothesis that the decellularized plant scaffold promotes long-term hemocompatibility and stable EC repopulation without the need for continuous pharmacological or cellular intervention. This is important because we previously demonstrated that endothelialization is required to prevent thrombosis of plant-based scaffolds in vitro [24]. EC density in the plant-based grafts was reduced by week 1 in vivo, but was significantly higher than that of silicone grafts at week 1 and week 4. Possible factors that may have contributed to the improved endothelialization of our plant-based grafts compared to the silicone grafts include natural biomimicry, hydrophilicity, and porosity. We previously demonstrated that decellularized plant scaffolds more closely match the elasticity of native vessels and promote EC attachment, even in the absence of a protein coating [23]. Most plant-based materials also have hydrophilic surfaces, which promote cell attachment better than synthetic materials like ePTFE, Dacron, and silicone. Additionally, the porous structure of plant scaffolds better supports tissue integration and EC infiltration compared to synthetic materials. All of these factors help maintain an endothelialized luminal surface that helps reduce thrombosis in vivo. Bacterial cellulose (BC) has previously been implanted in the carotid artery of rats for 1 year using shorter vascular grafts, 4 mm in length [48]. SEM revealed endothelialization of these grafts, but their limited length may reduce clinical applications compared to the 10 mm long grafts used in our study.

Patency and flow rates were monitored using Doppler ultrasound at key time points: 1, 4, and 24 weeks post-implantation. For the 1-week group, 17% of animals that received plant-based or silicone grafts survived the whole week. For these animals, successful integration with host vasculature and uninterrupted blood flow were observed. At 24 h and 72 h of the 7-day study, patency of the plant-based grafts remained high, with 67% and 50% remaining fully functional, while 33% and 17% of silicone grafts showed signs of reduced flow or occlusion. Additional cross-linked gelatin was applied for the 4-week study, increasing patency to 100% for plant-based grafts, compared to 67% for silicone grafts. At the 24-week point, 100% of rats implanted with plant-based grafts remained alive, with Doppler confirming blood flow, compared to 33% for silicone grafts. SEM imaging of these grafts revealed a smooth, thrombus-free luminal surface, closely resembling native aorta. In contrast, silicone grafts exhibited roughened inner surfaces and increased thrombus burden. Quantitative analysis of SEM images further supported these observations. Thrombus surface area fraction and thrombus width were significantly greater on silicone grafts at the 1-week time point, whereas plant-based grafts exhibited minimal thrombus burden throughout the study. Thrombus coverage and thickness on silicone grafts decreased at later time points, resulting in similarly low thrombus levels between groups by 24 weeks, likely reflecting thrombus remodeling, selective survival of less-thrombosed grafts, and design refinements implemented after early outcomes. These findings suggest that plant-based scaffolds not only maintain patency in the early postoperative period but also resist long-term thrombosis, likely due to their support of endothelialization and intrinsic hemocompatibility. Consistent with this interpretation, plant-based grafts exhibited improved short-term blood flow compared to the synthetic grafts. Early surgical outcomes informed subsequent refinements to graft design; following observation of early thrombosis and limited survival in non-reinforced grafts, later cohorts incorporated additional gelatin reinforcement to improve mechanical sealing and reduce postoperative bleeding, which was associated with improved survival at the 4- and 24-week endpoints. A limitation of this staged approach is that a gelatin-reinforced 1-week cohort was not included, precluding direct comparison of early outcomes between reinforced and non-reinforced grafts. SEM analysis provided further insight into these differences, revealing significantly increased surface roughness and thrombus formation in silicone grafts compared to plant-based grafts 1 and 4 weeks after implantation. Patency of the implanted plant grafts was lower than that of saphenous vein coronary bypass grafts in humans. When evaluating the remodeling process of implanted plant-based grafts and silicone grafts, significant differences in collagen and elastin deposition were observed over time. At week 1 post-implantation, H&E-stained sections revealed minimal tissue infiltration in both graft types, with no major structural changes indicating tissue integration. However, Picrosirius Red and Verhoeff-Van Gieson staining demonstrated that collagen and elastin coverage in plant-based grafts were comparable to those of the native aorta and significantly higher than those of silicone grafts. Quantitative analysis confirmed that the collagen content in plant-based grafts was comparable to that of the native aorta, remained stable at 1 and 4 weeks post-implantation, and increased slightly by week 24, which likely increased tensile strength but could also reduce compliance relative to native tissue. Conversely, collagen content in silicone grafts increased significantly 1 and 4 weeks after implantation. Elastin content in plant-based grafts and silicone grafts increased significantly by 4 weeks, then declined by 24 weeks. This suggests an initial healing response, where myofibroblasts lay down elastin fibers to restore compliance [49]. The subsequent decrease suggests that long-term elastin maintenance or new elastogenesis in the graft was limited. This phenomenon is consistent with challenges observed in other small-diameter grafts, where achieving stable, mature elastin in regenerated vessels remains difficult, as adult vascular cells rarely produce durable elastic fibers in vivo [50,51]. Early after injury, smooth muscle cell–rich neointima upregulates tropoelastin and deposits elastin, but the matrix is often immature and disorganized unless specific pro-elastogenic cues are present [52]. This likely explains the higher bulk elastin signal we observe at 4 weeks compared with 24 weeks. By 24 weeks, remodeling enzymes such as macrophage elastase MMP-12, which is secreted by macrophages and directly degrades arterial elastin, can reduce this early elastin deposition in the absence of sustained cues for elastic fiber maturation [53]. Importantly, no signs of intimal hyperplasia were observed in plant-based grafts, as shown by the absence of intimal layer thickening in VVG-stained sections. The 24-week plant-based grafts maintained a luminal wall area comparable to native aorta and silicone grafts, indicating that no significant stenosis or dilation occurred. This preservation of the lumen is a positive sign that the graft accommodates arterial flow without inducing pathological narrowing. The plant-based grafts exhibited a markedly thicker vessel wall compared to the native aorta. This increase in thickness may be attributed to swelling caused by the cross-linked gelatin in plant-based grafts. These findings suggest that plant-based grafts promote favorable remodeling with enhanced ECM deposition, integration with the host tissue, and minimal intimal hyperplasia, which could facilitate healthy vascular regeneration. Similar studies on biodegradable tissue-engineered grafts have demonstrated improved tissue integration but report complications such as rapid degradation and poor mechanical strength. In contrast, our plant-based grafts, with their endothelialized cellulose structure and layered vSMCs, minimized thrombosis and maintained structural integrity, striking a balance between bioactivity and material stability. At 4 weeks, the significant increase in collagen and elastin coverage in plant-based grafts further underscores their potential as a promising alternative for small-diameter vascular graft applications. Previous studies utilizing synthetic grafts such as ePTFE or Dacron have reported significant issues with intimal hyperplasia and thrombosis, often attributed to the inert nature of these materials and their lack of bioactive cues for endothelialization and tissue integration [54]. In contrast, our plant-based grafts exhibited more favorable remodeling outcomes with reduced incidence of intimal hyperplasia and reduced thrombosis, which suggests that these plant-derived materials may serve as a valuable alternative for promoting healthy vascular regeneration. Similar studies on biodegradable tissue-engineered grafts have also demonstrated improved tissue integration compared to purely synthetic materials [55]. However, in contrast to our findings, some studies have reported complications with rapid graft degradation or poor mechanical strength for cellulose-based scaffolds, leading to graft failure over time [56]. Our plant-based grafts, while minimizing intimal hyperplasia and thrombosis, maintained structural integrity throughout the observation period, likely due to the balance between bioactivity and material stability of our graft’s endothelialized cellulose structure layered with vSMCs. Importantly, the overall remodeling in the plant-based grafts appeared adaptive rather than pathological. The luminal surface remained smooth (indicating no excessive intimal fibrosis or narrowing), and there were no signs of calcification or aneurysmal degeneration. This suggests that the graft is remodeling in a manner that recapitulates a normal vessel wall, a conclusion supported by the presence of an organizing smooth muscle cell layer and newly deposited matrix by 24 weeks, akin to a neo-artery. Similar positive remodeling has been documented in other bioprosthetic grafts; for example, endothelialized silk fibroin grafts in rats showed a contraction of neointima and increased ECM maturation over time [57]. In our plant-based grafts, the increasing collagen content likely improved structural integrity and long-term durability, thus fulfilling a critical criterion of successful grafts. Meanwhile, the early presence of elastin, followed by a decline, highlights that further strategies may be needed to promote lasting elastic fiber formation for optimal compliance. Nevertheless, the ECM profile observed in the plant grafts at 24 weeks, increasing collagen and a maintained but modest elastin component, indicates a stable remodeling process rather than the maladaptive fibrosis typically seen in failing grafts.

Our 24-week results place plant-based vascular grafts in an encouraging position relative to other small-diameter graft technologies reported in the recent literature. The plant-based cellulose scaffold, reinforced with gelatin, combines features of natural ECM and a synthetic sealant, yielding a hybrid that performed impressively in vivo. Its ability to support rapid endothelialization and guided ECM deposition addresses two primary causes of synthetic graft failure: thrombosis and compliance mismatch. Recent studies on cellulose-based vascular scaffolds reinforce the promise of this material class. BC grafts, for instance, have shown excellent hemocompatibility and biostability in preclinical models. Fusco et al. reported that a 3 mm BC graft in a pig coronary bypass model was completely patent at 4 weeks, with a confluent endothelium and smooth muscle layer formation, evidencing in situ remodeling similar to our observations [16]. Longer-term studies of BC in sheep have demonstrated sustained patency in a subset of grafts up to 9–13 months, though overall patency rates ranged from 50 to 87% depending on antiplatelet therapy [48,58]. Compared to these outcomes, the high patency and EC density in our plant-based grafts at 6 months indicate that cellulose derived from plant tissue performs competitively.

A key advantage of the decellularized plant scaffold is its native vascular microarchitecture, which can guide cell migration and alignment to support organized tissue regeneration. Because mammals lack endogenous cellulases, cellulose-rich plant ECM is largely non-degradable in vivo, providing a persistent matrix for remodeling, an advantage over faster-degrading scaffolds that may fail before tissue maturation [59]. Consistent with this, BC vascular grafts remain intact for months with low inflammation while supporting endothelialization, including normal flow and patency in a five-month rat aortic replacement [60]. In a sheep carotid model, early BC prototypes showed approximately 50% patency at twelve weeks; patent grafts displayed confluent endothelial coverage with a vessel-like wall and no inflammatory signal, which is in line with cellulose’s slow degradation in vivo [61]. While off-the-shelf synthetic grafts like ePTFE or Dacron often remain acellular and exhibit poor 24-week patency (40%) and 3-year patency (25%), our plant-based grafts showed a significantly higher success rate at 24 weeks [62]. Although direct comparisons must be made cautiously, these findings suggest that decellularized plant ECM scaffolds may begin to approach the performance of autografts, which maintain patency rates > 90% in coronary bypass applications. Additionally, the acellular, xenogeneic yet low-immunogenic nature of these scaffolds makes them attractive for scalable, off-the-shelf applications without the complexity of human-derived or cell-seeded constructs.

The 24-week results support the potential of plant-based vascular grafts as clinically viable alternatives to synthetic materials. The grafts became endothelialized, maintained patency, and exhibited stable ECM remodeling without signs of inflammation or rejection, indicating strong biocompatibility and host integration. This integration suggests a reduced risk of late-stage complications like thrombosis or infection and underscores the benefits of a living endothelium for long-term patency. While collagen content increased over time, which may enhance strength, the observed decline in elastin highlights the need to address long-term compliance issues. Strategies to sustain or regenerate elastin through the delivery of precursors or biochemical cues may help maintain vessel elasticity and prevent stiffening. Importantly, the absence of immune-mediated rejection or chronic inflammation aligns with the known low immunogenicity of decellularized cellulose. The plant scaffold’s natural architecture also supports endothelialization, a feature lacking in synthetic conduits. From a translational standpoint, leatherleaf viburnum is inexpensive and readily available. The decellularization and gelatin cross-linking processes are simple and scalable, supporting the feasibility of off-the-shelf use [33]. These findings justify the next steps in larger animal models (sheep or pigs) to evaluate durability under physiological pressures beyond 6 months and assess risks such as calcification or aneurysm. Early preclinical success, combined with scalability and immune tolerance, positions plant-based scaffolds as promising candidates for future first-in-human studies in small-diameter vascular repair.

Inflammatory cell infiltration plays a central role in vascular graft healing and integration, and prior in vivo studies of plant-derived cellulose scaffolds have shown that macrophage-driven foreign body responses are generally mild and transient, resolving over time without evidence of chronic inflammation [63]. Early neutrophil recruitment is typically followed by monocyte-derived macrophages and lymphocytes, whose phenotype and persistence can distinguish constructive remodeling from chronic foreign body responses. While immunohistochemical identification of these cell populations provides valuable mechanistic insight, prior studies of plant-derived cellulose scaffolds have demonstrated favorable immune cell cytocompatibility in vitro, including improved white blood cell viability and reduced acute cytotoxicity following scaffold processing [25]. In the present study, effective decellularization, sustained endothelial coverage, and stable ECM remodeling together indicate a favorable tissue response at the graft interface.

To better understand the premature failure of grafts seeded with only ECs, we evaluated their permeability in vitro using a food dye perfusion test. This test revealed that grafts seeded with both ECs and vSMCs were able to withstand physiological pressures of over 15.8 kPa without any signs of leakage at 24 h, whereas grafts seeded with ECs alone began to leak at pressures of 10.3 kPa. The superior performance of grafts seeded with both cell types can be attributed to the role of vSMCs in providing mechanical support and structural reinforcement to the graft. vSMCs seeded onto scaffolds proliferate and deposit an ECM rich in collagen, elastin, and proteoglycans, which are key components found in native vessels [64]. Previous groups have concentrically wrapped polyglycolic acid and polycaprolactone polymer sheets seeded with fibroblasts, vSMCs, and ECs to achieve vascular grafts with a layered structure, but this method does not benefit from the natural architecture and biochemical cues found in natural ECM [65]. Blue food coloring was previously reported for leak experiments of vascular scaffolds, but it is unclear if physiological pressures were used [66]. vSMCs contribute to the strength of the vessel wall by replicating the natural architecture of native vessels, which contain both endothelial and smooth muscle layers [67]. This dual-layered structure helps our grafts maintain integrity under physiological pressure, preventing early leakage or collapse, which could explain their success in vivo. This highlights the importance of incorporating vSMCs in plant-based tissue-engineered vascular grafts to mimic the structure and function of native blood vessels more accurately, which rely on both cell types for mechanical stability and resilience under varying pressure conditions. EC-only plant grafts failed within one week, whereas constructs containing both cell types with gelatin reinforcement maintained integrity and achieved higher long-term patency. Replicating the medial layer’s structural and biochemical support appears critical for the success of plant-based vascular grafts.

Beyond barrier integrity, additional functional benchmarks such as vasoreactivity and endothelial nitric oxide–mediated signaling are often used to assess vascular graft maturation. Large-animal studies have demonstrated that tissue-engineered vascular grafts can evolve into neovessels capable of native-like physiological behavior, including flow-responsive remodeling and functional adaptation over time following implantation [68]. More recently, in vivo vasoreactivity testing has shown that implanted tissue-engineered grafts can exhibit active, reversible responses to vasoactive agents, supporting their functional integration into the circulation [69]. While such assays provide valuable insight into graft physiology, they typically require larger explanted tissue segments and specialized testing platforms and are therefore most often applied at later stages of graft maturation.

One limitation of this study is the use of the rat model, which, while valuable for early-stage preclinical research, presents differences in vascular physiology compared to humans. Rat arteries are smaller, and their hemodynamic, immune, and healing responses differ from those of humans. Additionally, immunohistochemical identification of inflammatory cell subtypes, such as macrophages and lymphocytes, was not performed, which would provide further mechanistic insight into the immune response associated with graft integration. The small caliber increases the risk of thrombotic occlusion and makes anastomoses more failure-prone, so patency in rats often represents a conservative estimate relative to larger-caliber human vessels. In clinical settings, larger diameters and routine antiplatelet therapy would be expected to favor patency, although comorbidities, longer graft lengths, and longer follow-up can offset these advantages. Consequently, translation should be validated in a large-animal model before extrapolating to clinical performance. Fluorescence-based identification of endothelial markers was not performed because plant-derived scaffolds exhibit strong intrinsic autofluorescence from lignin, chlorophyll, and polyphenolic compounds that overlap with the emission spectra of common fluorophores such as Alexa Fluor 568 and FITC, obscuring specific labeling. As demonstrated in our previous work, even after quenching treatments, residual autofluorescence persists and limits the reliability of immunofluorescence for these materials [27]. Our sample size was also limited to *n* = 3 per group, which, although typical for exploratory studies, reduces the statistical power and may limit the generalizability of the findings. Larger sample sizes for future studies would provide more robust data and help reduce variability.

## 5. Conclusions

The promising results from this study suggest that plant-based vascular grafts have potential for human applications, particularly in enhancing endothelialization and patency, which are key for small-diameter vascular grafts where synthetic materials often fail. However, the increased wall thickness observed needs further optimization to ensure long-term flexibility and prevent adverse effects like intimal hyperplasia in humans. Future studies should focus on larger animal models, such as pigs or sheep, to better mimic human vascular conditions and explore material modifications to balance bioactivity and mechanical properties. Early phase clinical trials will be essential to evaluate the safety and efficacy of these grafts in humans, potentially advancing the field of bioactive vascular grafts for clinical use. This study demonstrates that plant-based vascular grafts significantly enhance endothelialization, maintain high patency rates, and promote favorable remodeling compared to synthetic grafts in a rat model. These findings suggest that plant-based materials hold great potential for improving the performance of small-diameter vascular grafts and could play a crucial role in overcoming the limitations of synthetic grafts, offering new solutions for small-diameter vessel repair and reducing the risk of graft failure in clinical settings.

## Figures and Tables

**Table 1:** Mechanical and leakage testing results for decellularized leatherleaf scaffolds (2D) and vascular grafts (3D).

Property	Value
Tensile strength (N)	0.32 ± 0.09
Elastic Modulus (MPa)	2.98 ± 0.92
Suture retention (N)	0.85 ± 0.11
Burst pressure (mmHg)	409.8 ± 43.3
Compliance (%/100 mmHg)	3.41 ± 0.70
Leakage (mL/cm^2^/min)	0

**Table 2:** Summary of structural, morphological, and functional characteristics of plant-based vascular grafts compared to silicone grafts and native rat aorta.

Category	Feature	Plant-Based Graft	Silicone Graft	Native Vessel
Structure/Morphology	Base material/ECM	Decellularized plant cellulose scaffold reinforced with gelatin; supports mammalian cell adhesion	Synthetic silicone elastomer; non-ECM material	Native vascular ECM (collagen, elastin, proteoglycans)
	Wall thickness	Thicker wall due to plant scaffold and remodeling; stable over time	Thinner, uniform wall	Thin, compliant wall
	Collagen coverage/content	Comparable to native at 1 week; increased by 4 weeks; stabilized by 24 weeks	Low initially; increased deposition over time	Physiological baseline
	Elastin coverage/content	Present early; increased by 4 weeks; partial remodeling by 24 weeks	Minimal early; increased later	High, organized elastin
	Luminal surface morphology (SEM)	Smooth, continuous cellular coverage; low surface roughness	Rough, discontinuous surface with exposed material	Smooth, confluent endothelium
	Thrombus morphology (SEM)	Minimal thrombus; low surface area fraction and thickness	Substantial early thrombus burden; thicker deposits	No thrombus
Function/Performance	Endothelial cell coverage	High pre-implantation; recovers to native-like density by 24 weeks	Low and discontinuous at all time points	Continuous endothelial monolayer
	Thrombogenicity	Low early thrombosis; minimal thrombus burden over time	High early thrombosis; reduced only in surviving grafts	Non-thrombogenic
	Patency	Maintained patency through 24 weeks	Reduced patency and survival over time	Fully patent
	Hemodynamics (RI, PI trends)	Near-native resistance and pulsatility indices	Elevated resistance indices	Physiological
	Mechanical integrity	Adequate burst pressure, suture retention, and compliance for implantation	Mechanically stable but exhibits compliance mismatch	Optimized compliance and strength
Structure–Function Link	Overall interpretation	Biomimetic ECM, smooth luminal surface, and high EC coverage correlate with reduced thrombosis and sustained patency	Lack of ECM and rough surface correlate with thrombosis and reduced patency	Structure inherently optimized for function

## Data Availability

The raw data required to reproduce these findings will be available to download from IEEE DataPort at https://doi.org/10.21227/5q0x-xz93 upon publication (accessed on 12 January 2026).

## Supplementary Materials

The following supporting information can be downloaded at: https://www.mdpi.com/article/10.3390/jfb17010043/s1, Figure S1. Illustration of fabrication process for plant-based graft. SDS-decellularized leatherleaf is seeded with vascular smooth muscle cells and rolled counterclockwise onto a 1.5 mm mandrel. The orange represents the addition of gelatin and the blue circles are glutaraldehyde. Figure S2. Food coloring permeability tests of (a) cell-seeded plant-based grafts, and (b) 2D leatherleaf sheet setup. Figure S3. Surgical setup: (1) pulse oximeter, (2) light source, (3) nose cone, (4) heating pad, (5) induction chamber, (6) surgical microscope. Figure S4. Vascular graft ultrasonography showing (a) typical grayscale B-scan of implanted graft (solid yellow arrow) showing hyperechoic feature (solid blue arrow) due to reflector used as a graft locator guide, (b) associated Doppler image showing blood flow in implanted graft (solid yellow arrow) with graft diameter measurements and a preset Doppler region of interest (thin yellow box) as pointed by the hatched yellow arrow, (c) region of interest (ROI) manually selected in the area adjacent to the graft site (solid yellow arrow), and (d) dithered binary map version of the image within the ROI shown in C used to determine the heterogeneity index (HI). Figure S5. Images of histology for H&E of (a–c) plant grafts, (d–f) silicone grafts, and (g) aorta, 1, 4, and 24 weeks after implantation at 4× magnification.

## Abbreviations

The following abbreviations are used in this manuscript:

ECM	Extracellular matrix
EC	Endothelial cell
vSMC	vascular smooth muscle cell
SDS	sodium dodecyl sulfate
LOQ	limit of quantification
RI	resistive index
PI	pulsatility index
HI	heterogeneity index
ROI	region of interest
SEM	scanning electron microscopy
