# Supplementary material for: Implantation of Bioreactor-Conditioned Plant-Based Vascular Grafts
Source: J Funct Biomater. 2026 Jan 15;17(1):43. doi: 10.3390/jfb17010043 (PMC12843382; doi:10.3390/jfb17010043)
Supplement: Supplementary file 1 [file jfb-17-00043-s001.zip › jfb-4055596-supplementary.pdf]

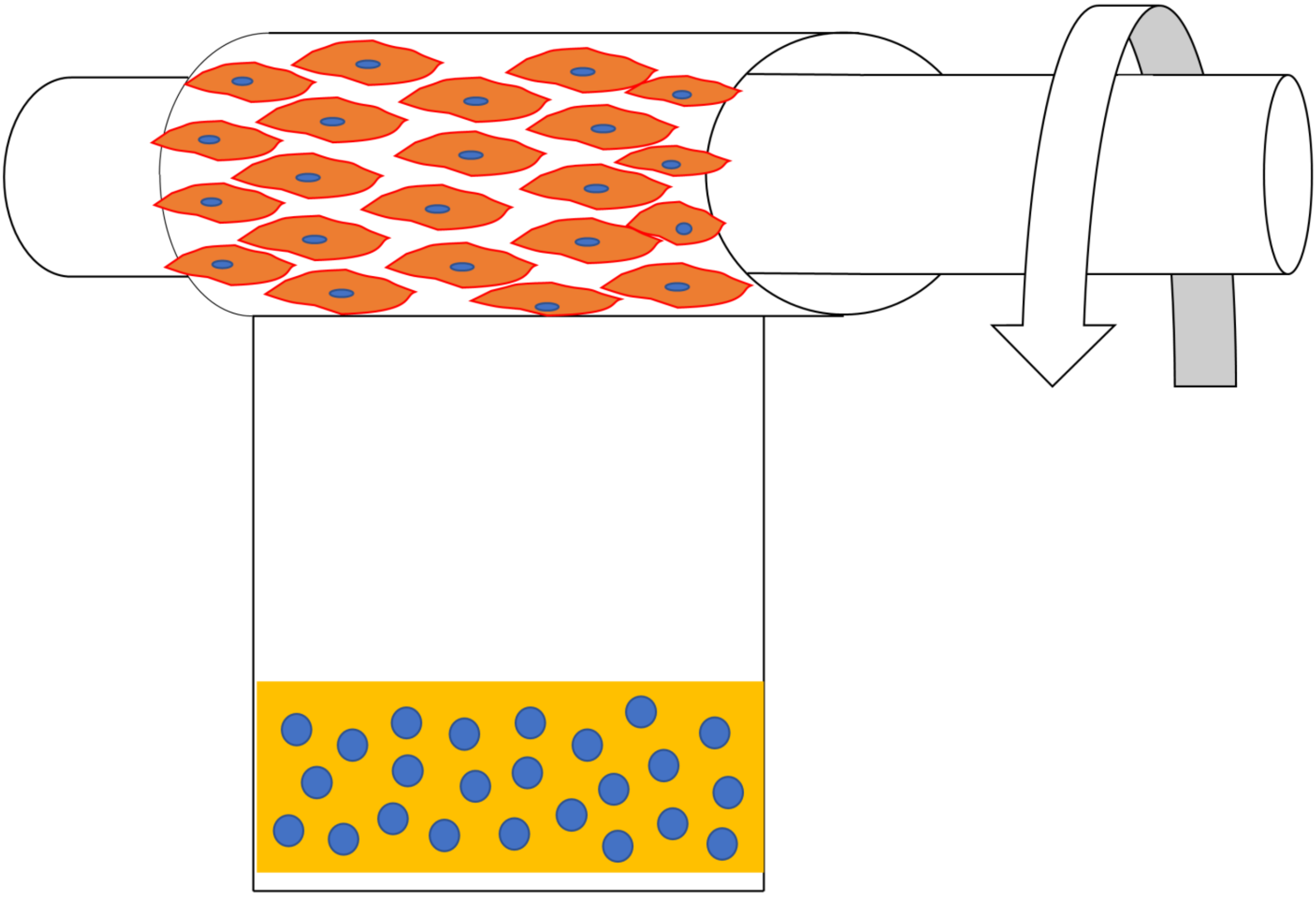

Supplemental Figure S1. Illustration of fabrication process for plant-based graft. SDS-decellularized leatherleaf is seeded with vascular smooth muscle cells and rolled counterclockwise onto a 1.5 mm mandrel. The orange represents the addition of gelatin and the blue circles are glutaraldehyde.

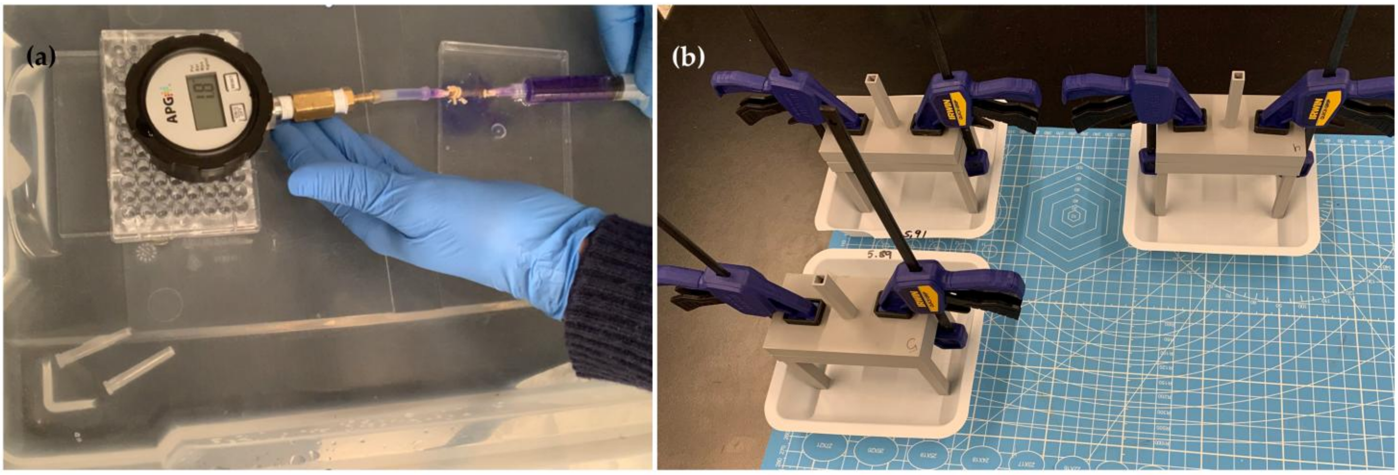

Supplemental Figure S2. Food coloring permeability tests of (**a**) cell-seeded plant-based grafts, and (**b**) 2D leatherleaf sheet setup.

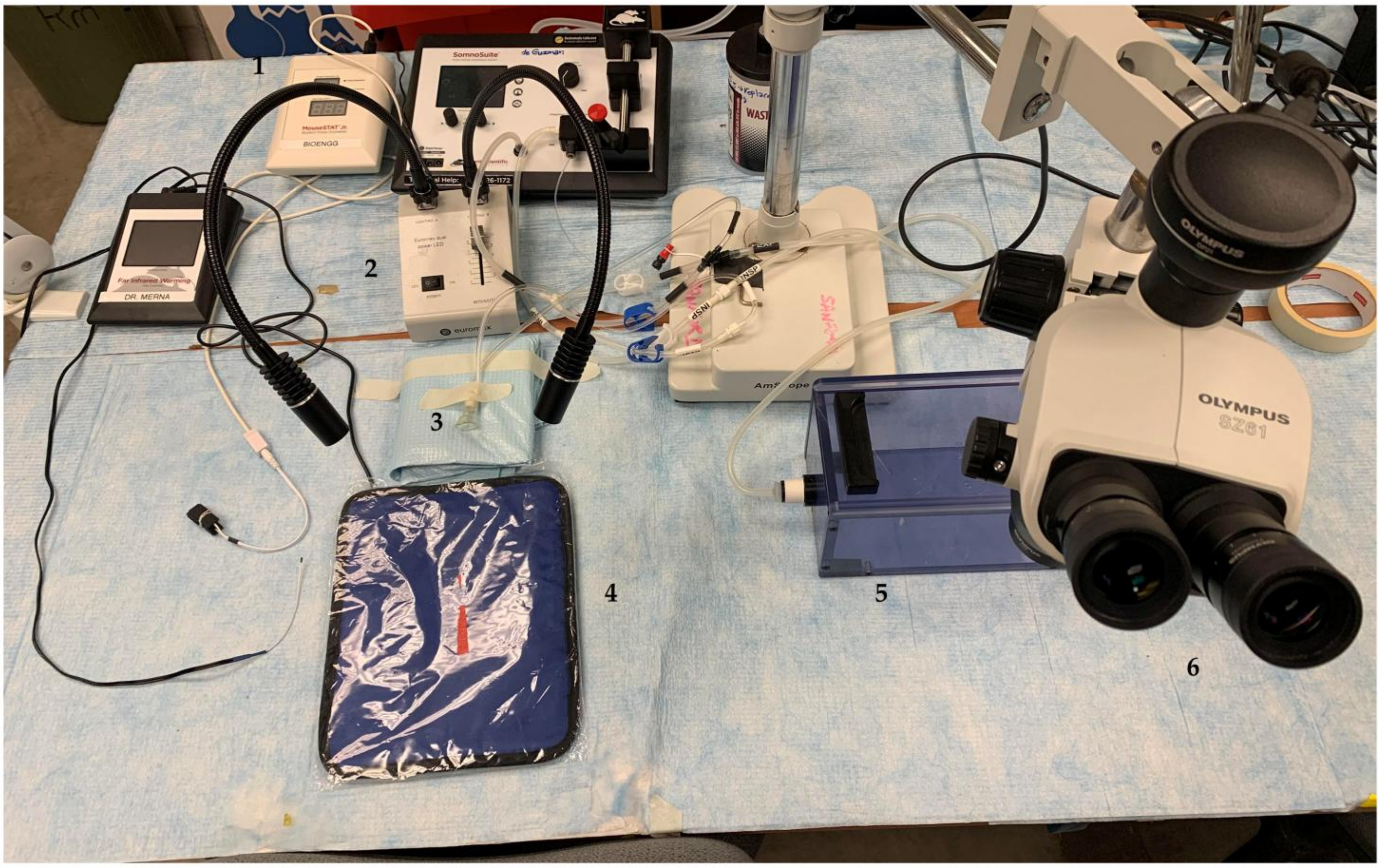

Supplemental Figure S3. Surgical setup: (1) pulse oximeter, (2) light source, (3) nose cone, (4) heating pad, (5) induction chamber, (6) surgical microscope.

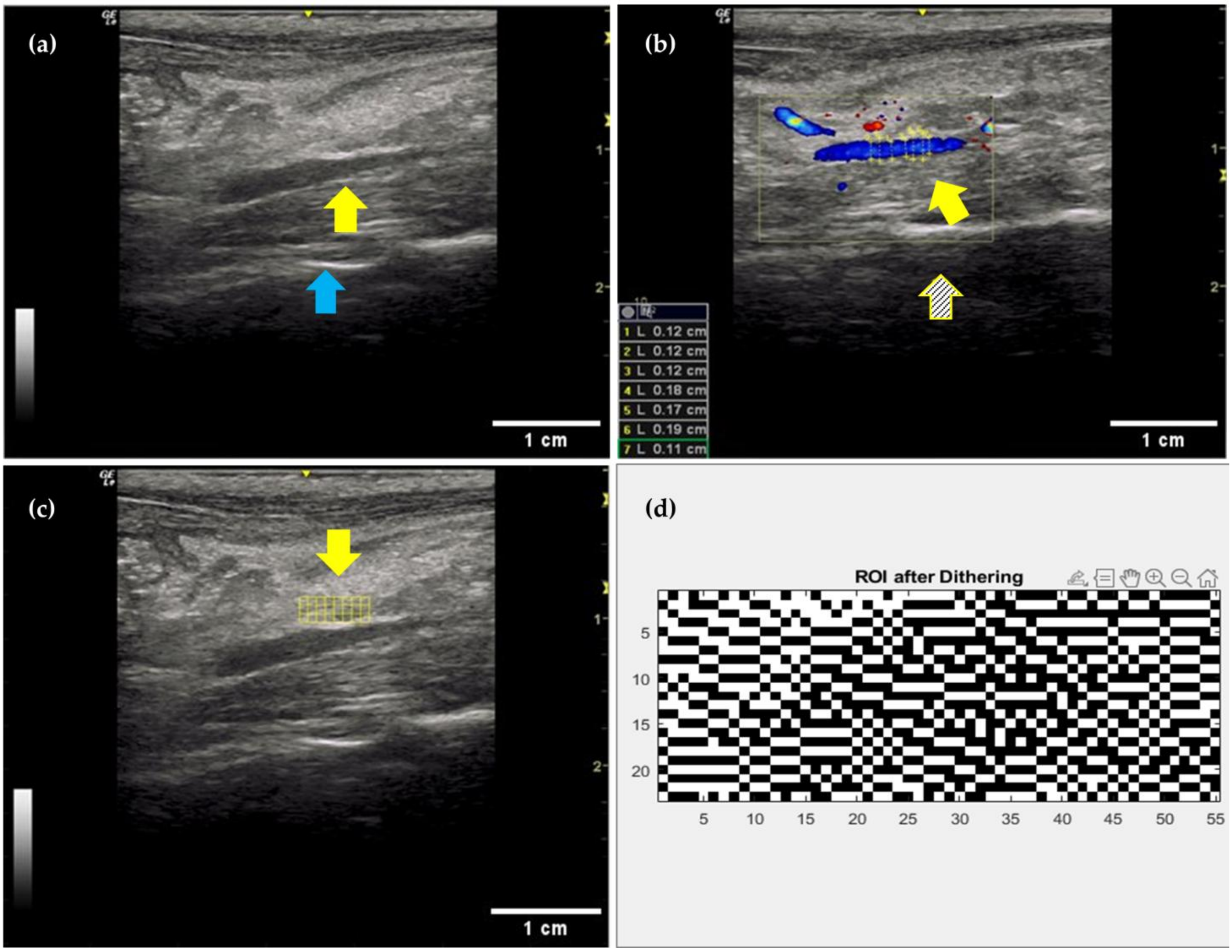

Supplemental Figure S4. Vascular graft ultrasonography showing (**a**) typical grayscale B-scan of implanted graft (solid yellow arrow) showing hyperechoic feature (solid blue arrow) due to reflector used as a graft locator guide, (**b**) associated Doppler image showing blood flow in implanted graft (solid yellow arrow) with graft diameter measurements and a preset Doppler region of interest (thin yellow box) as pointed by the hatched yellow arrow, (**c**) region of interest (ROI) manually selected in the area adjacent to the graft site (solid yellow arrow), and (**d**) dithered binary map version of the image within the ROI shown in C used to determine the heterogeneity index (HI).

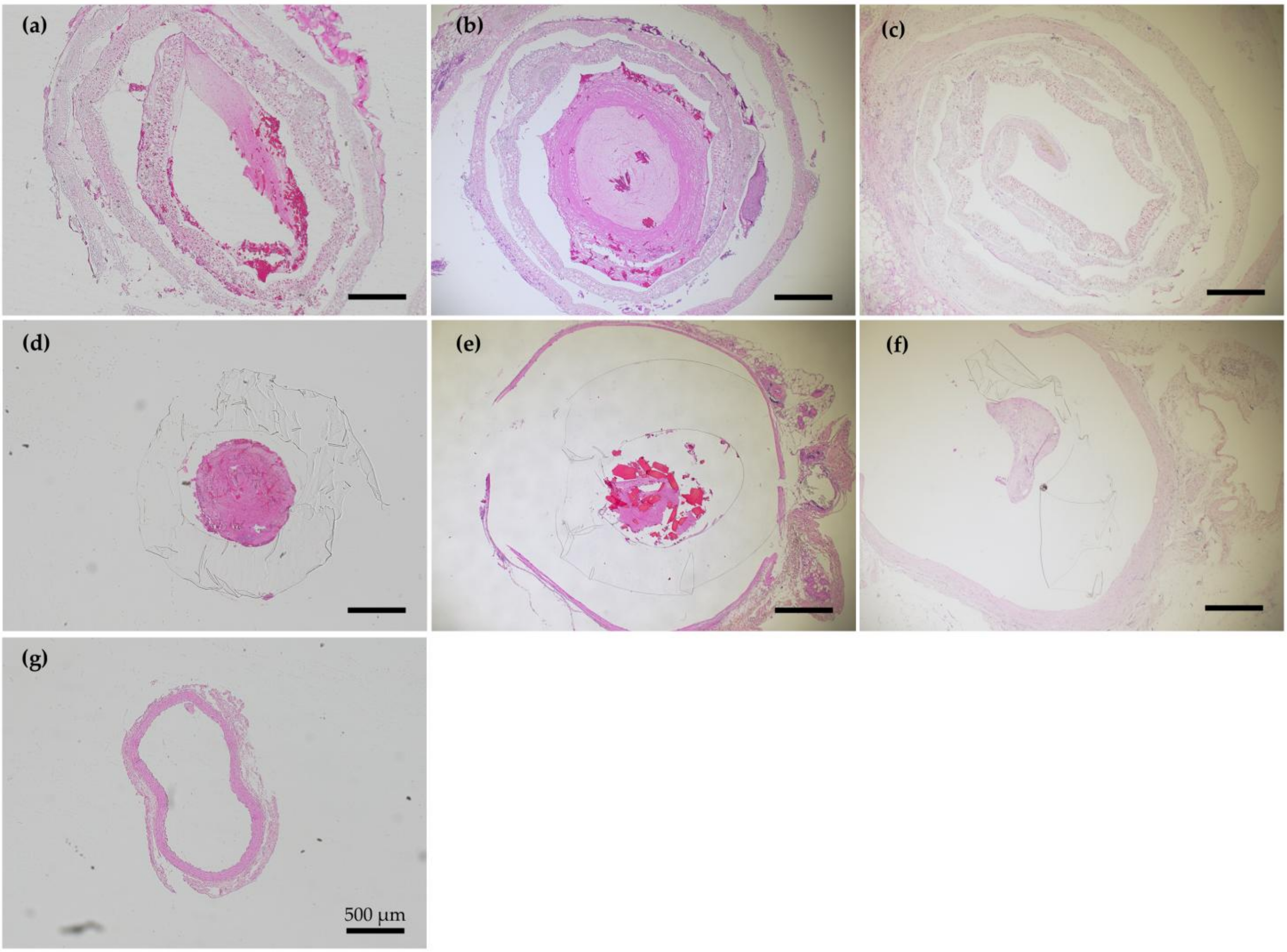

Supplemental Figure S5. Images of histology for H&E of (**a-c**) plant grafts, (**d-f**) silicone grafts, and (**g**) aorta, 1, 4, and 24 weeks after implantation at 4× magnification.
